# Cultural and Human Capital Signals in Hiring—A Factorial Survey Experiment Across Contexts

**DOI:** 10.1111/1468-4446.70125

**Published:** 2026-05-20

**Authors:** Luisa Burchartz, Kobe De Keere, Sara Geven

**Affiliations:** ^1^ Department of Language, Literature, and Communication Utrecht University Utrecht the Netherlands; ^2^ Sociology Department University of Amsterdam Amsterdam the Netherlands

## Abstract

When evaluating candidates, hiring agents may draw on signals of human as well as cultural capital. While these processes have been considered separately, an open question is how the two types of signals interact. As signals of social class, cultural capital signals relate to human capital as they evoke stereotypes about competence, polish, and discipline, and can indicate different patterns of social mobility in combination with human capital signals. These interdependencies may be important in employee gatekeeping, and therefore in the reproduction of labour market inequalities. In this paper, we address the question of how hiring agents evaluate cultural capital signals in light of different human capital signals. We draw on a factorial survey experiment to analyse hiring agents' evaluations of different cultural capital signals (combining signals of class backgrounds and class practices) in relation to human capital indicators (the level and specificity of education, training participation, and work experience) across contexts. These contexts include two countries, Germany and England, two sectors, Software Development and Human Resource Management, and two career stages, the early and late career. The results show that the value of highbrow cultural capital signals depends on the context, as well as on human capital signals. In Software Development and HRM as the professional fields that we studied, hiring agents often did not give value to cultural capital signals. This was especially the case in England. In Germany, positive evaluations of cultural capital signals mostly followed a multiplication effect: such signals were only valued when higher human capital signals were also indicated. We found this pattern most clearly in Germany, particularly for late‐career candidates. This multiplication effect underlines the importance of looking at combinations of cultural and human capital signals for understanding how inequalities are generated in gatekeeping processes.

## 1 Introduction

As hiring processes are moments of gatekeeping and sorting, studying them is key to increasing our understanding of how labour market inequalities come about (Bills et al. 2017; Rivera 2020). While human capital and productivity have long taken the centre stage in how social scientists have thought about employers' decisions, the *Sociology of Evaluation* highlights that hiring agents draw on a variety of criteria, often simultaneously (Lamont 2012). These include status beliefs and stereotypes, emotional energy, homophily and fit, personality traits, and cultural scripts and ideals about merit and workers (Ashley and Empson 2017; De Keere 2021; Friedman and Laurison 2020; Hamann and Beljean 2021; Hora 2020; Moss and Tilly 2001; Neely 2018, 2020; Nichols et al. 2025; Rao and Neely 2019; Ren 2024; Sølvberg and Rivera 2025; Tholen 2023).

Particularly in high‐status professions, where hiring agents can choose between many different candidates who all have elite qualifications, signals of social class backgrounds are key criteria to distinguish between candidates. As signals of social class, cultural capital signals create labour market advantages as they evoke positive stereotypes of competence and polish, as well as feelings of familiarity among evaluators who share upper‐class lifestyles (Rivera 2015; Rivera and Tilcsik 2016; Thomas 2018). Cultural capital, as also Bourdieu (1984) described it, becomes the central currency in the allocation of resources in elite fields.

In the wider labour market beyond elite professions, however, pools of potential candidates are much more varied. Hiring agents have to make trade‐offs between candidates who have the right qualifications (e.g., a Bachelor's degree in the specific field or have done recent training) and someone who fits with a middle‐class lifestyle. Here, hiring agents may have to negotiate criteria related to both human^1^ and cultural capital signals.

Whereas prior research documents the context dependency of the currency of cultural capital signals on countries, sectors, on who evaluates, and other candidate characteristics (De Keere 2021; Friedman and Laurison 2020; Nichols 2023; Noordzij et al. 2024; Reeves and De Vries 2019; Ren 2024; Rivera and Tilcsik 2016; Streib 2023; Thomas 2018), this dependency of the value of cultural capital on human capital has been largely neglected in the literature. In this paper, we address this gap; looking at how hiring agents evaluate classed cultural capital signals in relation to different human capital signals. We specifically look at cultural capital signals as combinations of signals of class backgrounds and cultural practices.

The dependence between cultural and human capital signals is important as, from a Sociology of Evaluation perspective, we can expect clear contingencies. Class backgrounds not only affect educational outcomes, implying that higher cultural capital tends to be paired with higher human capital signals; but highbrow cultural capital signals also create stereotypes around competence, polish, and discipline (Ren 2024; Rivera and Tilcsik 2016; Thomas 2022), attributes that are intertwined with perceptions of skill and competence. Candidates with years of experience in the sector and a targeted educational profile may be able to complete the picture of the ideal candidate when they also match upper‐(middle)‐class norms. For instance, when they have the right taste in music. These candidates may also be seen as especially apt to (re)present products and knowledge. Moreover, combinations of signals of social class backgrounds and human capital signals may create images of social mobility which hiring agents may value as meritocratic life stories, or devalue as signs of unwarranted privilege (Rivera 2015).

In addition to prior research on cultural capital, social stratification theory on the interplay of different kinds of capital in life‐courses, too, gives insights into potential mechanisms (DiPrete and Eirich 2006; Erola and Kilpi‐Jakonen 2017). First, advantages may multiply: having more of different forms of capital may create even more capital. Moreover, we can also imagine patterns of compensation: having relatively more of one form of capital may create possibilities to compensate for a lack of other forms of capital. While research on the contingency between cultural and human capital signals has been limited, there is evidence for both scenarios. Two studies raise the expectation that high cultural capital only leads to rewards in the presence of indicators of high human capital (Jackson 2009; Reeves and De Vries 2019). Using survey data, Reeves and De Vries (2019) examine the odds of promotions, pay raises, and upward mobility in the UK. They show that employees only benefit from highbrow cultural consumption if they have university qualifications. Second, Fossati et al. (2020) suggest that the signalling of more upper‐class parental occupations (but not leisure activities) can compensate for unclarity in human capital signals on the Swiss apprenticeship market.

In this paper, we combine insights from research on cultural capital and stratification research through a sociology of evaluation lens (Lamont 2012). This evaluation perspective also highlights the importance of contexts for how evaluations are being done. Here, we examine the interactions of cultural and human capital signals by focusing on hiring agents' evaluations in three contextual spheres, which we approach as case studies. These include different career stages (early and late), sectors (Software Development and Human Resource Management [HRM]), and countries (Germany and England). The career stages differ on potentially different status and skill expectations across the career (Burchartz 2025; Friedman and Laurison 2020; Meyer 1977). The middle‐class sectors of HRM and Software Development represent a setting where we can expect cultural capital to be valued, while a variety of human capital signals may be acceptable. Finally, in both countries, cultural capital has been shown to matter in Bourdieusian ways, while differing in class identification patterns and in how skills tend to matter in the labour market (Busemeyer 2009; Crouch 2006; D’Hooge et al. 2018; Di Stasio and Van de Werfhorst 2016; Oesch and Vigna 2023; Thelen 2004). The goal of our study, however, is not primarily comparative. Instead, it is to examine how the interaction between cultural and human capital is context‐dependent, thereby illuminating how sectors, career stages, and countries shape the role these evaluative criteria play in hiring decisions.

Methodologically, we draw on a factorial survey experiment, where we asked hiring agents to evaluate fictional candidates who differ in their cultural and human capital profiles. Here, we indicated cultural capital signals through combinations of class backgrounds (names) and cultural tastes (leisure activities). This approach allows us to directly test employers' judgements as a mechanism (e.g., rather than observing promotion patterns through survey data) that contributes to the reproduction of inequalities on the labour market. Although this approach comes with challenges (e.g., targeting a very narrow population), it allows for disentangling the separate impact of cultural and human capital signals on evaluations. This would be difficult with observational (survey) data, as human and cultural capital signals are highly correlated in the real world.

We contribute to the current literature in the following ways: we systematically look at interactions between human capital signals and cultural capital signals in the evaluations of hiring agents, examining four human capital indicators that capture various human capital facets, including the level and field of education (as education specificity), work experience, and on‐the‐job training. Further, we study the evaluations of cultural and human capital across countries, sectors, and career stages. The focus on two country cases especially differs from prior literature and adds an additional contribution. This is relevant as the labour market value of cultural capital signals has often been studied in more liberal market economies (like the UK or the US) and less in countries like Germany, where skill formation typically revolves around specific educational credentials.

The results emphasise the context dependency of the labour market value of (highbrow) cultural capital signals, also on human capital signals. In the professional fields of Software Development and HRM that we have studied, hiring agents often did not attribute value to cultural capital indicators shown in profiles, especially in England. When they did, the value of cultural capital signals mostly depended on the presence of human capital signals. This appeared as a multiplication effect: evaluators valued highbrow cultural capital signals (more) when high human capital was also signalled. We most clearly observe this pattern in Germany, especially in the late career.

## 2 Theoretical Framework

### 2.1 Evaluations of Cultural Capital Across Contexts

Hiring processes necessarily include making sense of and evaluating signals (Spence 1973). As the evaluation perspective points out, hiring agents do not only draw on human capital signals to gauge productivity, but they draw on a variety of signals and criteria. They consider different types of capital (human, social and cultural), as well as a variety of signals about candidates' belonging to status groups, including signals of gender, race/ethnicity, religion, and class backgrounds (for an overview, see Rivera 2020) and they evaluate these signals according to different criteria (Bills et al. 2017). Next to productivity, they, for instance, also value passion, cultural fit, emotional energy, and evaluate candidates according to worker ideals.

In this paper, we focus on cultural capital signals as one signal that can play a crucial role in labour market allocation (Friedman and Laurison 2020; Rivera 2015). We understand cultural capital as the ‘possession of legitimate [defined by the dominant class] knowledge, skills and tastes’ (Friedman and Laurison 2020, 14), which ‘always remains marked by its earliest conditions of acquisition’ (Bourdieu 1986, 18). According to this line of reasoning, cultural capital signals necessarily connect the current class positions to people's class backgrounds (Bourdieu 1984; Lamont and Lareau 1988). In application procedures, class backgrounds and cultural capital signals are observable through names, leisure activities, educational qualifications, and even subtler cues like aesthetics in application materials (Rivera 2015).

Highbrow cultural capital cues shape impressions in a twofold way by (1) locating the person in the social space and (2) evoking stereotypes (Thomas 2022). The former involves a subtle, often subconscious sense of the social class background of the other. Think of the classic example of ‘visiting art museums’ as a hobby that signals a high cultural capital background (Bourdieu 1984). This process does not only point hiring agents to candidates' current class positions but to more long‐term biographical practices. Cultural capital signals tend to carry information about candidates' upbringing. Moreover, these signals, and the positions that are associated with them, also evoke stereotypes about candidates' abilities and behaviours. Concretely, highbrow signalling is associated with competence and polish and, to some extent, with discipline and control (Ren 2024; Rivera and Tilcsik 2016; Thomas 2018). Polish refers to (upper‐)middle‐class self‐presentation, dressing in the right way, using the right words, and making the right jokes. To some extent, however, highbrow signals are also linked to negative stereotypes: people with higher cultural capital signals can be considered colder (Rivera and Tilcsik 2016), arrogant, smug, or snobbish (Jarness and Flemmen 2019).

Despite the widely positive stereotypes, research suggests that cultural capital signals are not a universal currency (Erickson 1996), but that other ascribed characteristics and sectors influence how cultural capital cues play out in hiring processes. Candidates' gender, for instance, alters the stereotype content of cultural capital signals and which criteria of evaluation are most scrutinised in evaluations (Rivera 2015; Rivera and Tilcsik 2016; Thomas 2018). Further, fields differ in how highbrow cultural capital signals are valued and rewarded (Friedman and Laurison 2020; Nichols 2023; Reeves and De Vries 2019; Streib 2023). This is partially due to cultural matching, as cultural signals produce advantages by creating feelings of homophily, clicking, and emotional energy (Rivera and Tilcsik 2016). While high‐cultural capital signals provide grounds for matching in elite settings, in more class‐diverse, or relatively lower‐class contexts, the value of highbrow cultural capital is less evident (Erickson 1996; Lamont 2000; Nichols 2023; Noordzij et al. 2024; Reeves and De Vries 2019; Streib 2023). In non‐elite settings, candidates' variety in tastes and consumption may instead be more beneficial (Erickson 1996).

Field‐specific worker ideals also impact the value of cultural capital (Friedman and Laurison 2020). What is considered a ‘good’ or ‘ideal’ worker is shaped by historically dominant groups within fields, thereby differing along the lines of gender, racial categories, and class backgrounds (Ashcraft 2013; Ashley and Empson 2013; Friedman and Laurison 2020; Hora 2020). Examples of profession‐specific worker ideals include ‘nerdy’ young men in Software Development (Ensmenger 2010) and upper‐class men in finance (Ho 2009). Often, these ideals are classed, which then turns cultural capital into a hiring criterion in the search for alignment (Ashley and Empson 2013). As job‐specific meanings of skills and competence are ingrained in these worker ideals, the social class dimension and understandings of merit and skill become intertwined (Blair‐Loy and Cech 2022). This is particularly pronounced in interaction‐intensive fields, where the value of polish is amplified (Thomas 2018), and in knowledge‐ambiguous professions. In these knowledge‐ambiguous professions characterised by low levels of standardisation (of operations, processes, and criteria to evaluate performance), appearance, speech and mannerisms, and with it, cultural capital, become rather prominent judgement criteria (Alvesson 2001; Ashley and Empson 2013; Friedman and Laurison 2020; van den Berg and Arts 2019).

### 2.2 Cultural and Human Capital Signals

Cultural capital signals are intertwined with stereotypes about skills, learning, and competence, as well as with conceptions and meanings of ‘skills’ through worker ideals. Simultaneously, hiring agents' making sense of cultural capital signals also includes locating candidates in the social space and thereby, forming some (implicit) understanding of social class backgrounds. In combination with human capital signals, such as the possession of a university credential, these interpretations may create images of class trajectories.

While we have discussed cultural capital above, we have thus far paid little attention to human capital. In this paper, we consider four common human capital cues, including the level (vocational training or university degrees) and specificity of education, work experience, and on‐the‐job training. These capture different facets of human capital: they include more general and transferable skills (applicable across occupations), job‐specific skills; differences in the length and intensity of skill acquisition, differences in when the skills have been acquired, and in the setting of acquisition (formal education, workplace, training provider) (see e.g., Becker 1962). Of course, university qualifications go beyond signalling human capital (Bills 2003). They simultaneously indicate social class or cultural capital (Collins 1979) and, from a neo‐institutionalist perspective, categorise people as university graduates (Meyer 1977; Tilly 1998). This categorisation can then legitimise hierarchies and expertise. Instead of disentangling different dimensions of human capital, however, we look at human capital cues as a more unified concept in evaluations of hiring agents in this paper.

To think about the interaction of cultural capital and human capital, we draw on two mechanisms formulated in social stratification research: multiplication and compensation (DiPrete and Eirich 2006; Erola and Kilpi‐Jakonen 2017).

From the multiplication perspective, agents may especially reward candidates who signal high cultural and high human capital (DiPrete and Eirich 2006). Reeves and De Vries' (2019) study on the British labour market suggests that employees only benefit from cultural consumption if they have university qualifications and not at other levels of education (e.g., further education, GCSEs, or an apprenticeship). Cultural capital signals may only become a relevant evaluation criterion if human capital requirements are also met, to distinguish between profiles that are seen as well‐suited and/or equivalent concerning human capital signals. Multiplication patterns could also result from the competence signals of job‐specific skills and cultural capital enhancing each other. The embodied competence associated with cultural capital may be considered especially valuable when combined with evidence of stronger job‐specific skills; or cultural capital may be viewed as necessary to convey job‐specific skills.

Next, cultural capital signals can potentially compensate for deficient or ambiguous human capital signals (Erola and Kilpi‐Jakonen 2017). The relative benefits of cultural capital signals may be especially high for candidates with lower or unclear human capital signals, as suggested for parental occupation as a status signal in prior research on the Swiss apprenticeship market (Fossati et al. 2020). By evoking positive class‐based stereotypes around competence and discipline, these candidates could be considered able to make up for a perceived deficiency or ambiguity in human capital. While strong human capital signals, such as educational qualifications or extensive work experience, are unlikely to be offset by cultural capital signals alone, other, less formative signals (e.g., on‐the‐job training) can potentially be compensated for.

As suggested above, combinations of cultural and human capital signals may suggest particular social mobility patterns (upward or downward). This would particularly be the case when hiring agents interpret signals in light of social class *backgrounds*. Hiring agents may value or penalise such patterns, especially in societies with strong meritocratic belief systems.^2^

The most likely scenario is the rewarding of combinations of high human capital and signals of lower social class backgrounds. Relative to the candidates that signal privileged class backgrounds, those who indicate lower class backgrounds may be viewed as having achieved more than would typically be expected; they could then be seen as more competent and hard‐working (Reeves and Friedman 2024). Moreover, Rivera (2015) describes that hiring agents value storytelling in hiring processes. Again, where meritocratic stories are viewed as valuable, upward social mobility patterns may be considered valuable. Following a similar logic, combinations of signals of upper‐class backgrounds and lower human capital signals may be penalised as downward mobility trajectories. Such candidates could be perceived as violating class norms around university attendance and human capital acquisition. Moreover, as upper‐class people are sometimes viewed as ‘lucky’ to ‘have landed on the right side of things’ (Jarness and Flemmen 2019, 174), their human capital acquisition may be seen as comparatively straightforward. Hence, not collecting the low‐hanging fruit may be interpreted as laziness and/or incompetence.

## 3 Contexts

We look at the evaluations of cultural and human capital signals at (1) different moments in the career, namely in the early and late career; (2) in two sectors–HRM and Software Development, and lastly, (3) in two countries–Germany and England.

### 3.1 Career Setting

We look at the interaction between the two types of signals in the early and late career. Prior research suggests that evaluations of cultural and human capital signals are not uniform across the career, which means that the interactions between the two may also vary. Friedman and Laurison (2020, 4) find a growing importance of class backgrounds throughout the career. Even after entering elite occupations, lower‐class backgrounds create additional barriers, and upper‐class backgrounds a ‘following wind’ that spurs career progression. One reason is that worker ideals associated with being partner material require more polish than at career entrance. For human capital, Sharone (2024) depicts the difficult experiences of older, highly qualified job seekers: when applying to lower‐level jobs, these workers tend to be viewed as too skilled and too expensive, coupled with the expectation that they would quickly be bored, resulting in high turnover. A positive stereotype about older workers is that they have better management skills (Karpinska et al. 2013).

Older workers then tend to be associated with more senior positions and responsibility within organizations. Such ‘expanded social roles’ come with increased status expectations, tied to university credentials (Meyer 1977, 62), Here, candidates usually have to demonstrate tenure within the field, alongside status and polish to demonstrate leadership skills (Burchartz 2025; Friedman and Laurison 2020). For these roles, cultural capital signals can play a larger role (Burchartz 2025; Friedman and Laurison 2020), while also being tied to the institutional status expectations of university education (Meyer 1977), and relevant work experience (Sharone 2024).

Also, the literature on age discrimination shows that a key stereotype against older workers is a lack of trainability, meaning that they are perceived as having difficulties in acquiring new skills (Karpinska et al. 2013). In terms of the mechanisms mentioned above, this importance could, for instance, make it harder to compensate for on‐the‐job training in later careers.

### 3.2 Sector Setting

The study focuses on the cases of Software Development and HRM as sectors where we may expect cultural capital signals to play a role, while different human capital profiles can be considered. Both are typically classified as professional fields and knowledge work; and they certainly are middle‐class professions. While they do not represent an elite field with very high human capital expectations (like an elite university credential) where cultural capital then becomes central, it is still reasonable to expect cultural capital to matter in this middle‐class, knowledge work fraction of the labour market. Based on Bourdieu's (1984) arguments in Distinction, one could even go as far as to suggest that cultural capital signals may matter especially in the middle class. For those with relatively less capital (in comparison to the upper‐class), the potential of social mobility creates cultural goodwill, the makingup or compensating for a relative lack of knowledge by demonstrating recognition and legitimacy.

Next to these theoretical expectations regarding cultural capital, the professions share a lack of credential or licence requirements. Compared to occupations like medical doctors, where practitioners need to have obtained specific credentials (and/or licences), anyone could theoretically be hired for HR and Software positions, regardless of their education. This means that hiring agents can consider profiles with differences in human capital profiles. We could then think of these cases as (most‐)likely cases to observe interactions between human and cultural capital signals (Gerring 2009).

Considering middle‐class, knowledge work professions without regulatory credential requirements, the Software Development and HRM then differ on three key dimensions that have been shown to shape the value of cultural capital in the labour market. These include worker ideals, the degree of knowledge ambiguity and interaction intensity.

In Software Development, there are gendered^3^ and age‐related worker stereotypes (around nerdy young men or boys), combining attributes around being clever, antisocial, and eccentric (Ensmenger 2010). While usually classified as knowledge management, the work also includes elements of manufacturing and craftsmanship (Marks and Scholarios 2007) and is historically taught in academic education and apprenticeships (Ensmenger 2010). Further, evaluations of skills and knowledge and definitions of expertise are rather clear‐cut and based on shared and delineated criteria (Burchartz 2025). In contrast, HR Management is mostly practiced by women^4^ and combines both female‐coded notions around communication and (emotional) support, as well as male‐coded, managerial ambitions (Ainsworth and Pekarek 2022). The historically rooted managerial traditions also emphasise the upper‐(middle‐)class location of the HR profession. Considering this emphasis on communication, support, and management skills, the criteria for evaluating knowledge in the field are fairly ambiguous. This ambiguity comes with an emphasis on naturalised notions of competence and personality in the skill evaluations of applicants (Burchartz 2025). Based on the managerial tradition, knowledge ambiguity, and interaction intensity, cultural capital signals would be expected to play a prominent role in HRM, and less so in Software Development.

### 3.3 Country Setting

We further conducted the experiment in two European countries: Germany and England. While research on cultural capital in Germany remains limited, studies in both countries have demonstrated the importance of cultural capital (signals) in perpetuating inequalities (Hartmann 2000). This also may stem from relatively similar volumes and composition of different types of capital at the national level (economic, military, symbolic, political, and cultural‐technical) in global comparison (Atkinson 2022). In comparison to other countries like China, where cultural capital signals do not play out how we typically expect, but where sports were particularly valued as a sign of stamina and resilience (Ren 2024), we decided that the general applicability of Bourdieusian frameworks is a crucial criterion of case selection. Additionally, key differences that make the two countries interesting cases to look at lie in how people in Germany and England relate to social class and in different skill formation regimes.

In the political economy literature, Germany and England are considered prototypes of different skill formation systems (Busemeyer 2009; Crouch 2006; Thelen 2004). In Germany, where employers have historically been involved in shaping the curricula in educational institutions, vocational and specific education are usually valued very highly by employers. In this setting, notions of *being skilled* are tied to having specific credentials (Thelen 2004). In England, the workplace is usually considered the place to learn job‐specific skills (Busemeyer 2009; Crouch 2006). Therefore, hiring agents put relatively more emphasis on the ability and willingness to learn (Di Stasio and Van de Werfhorst 2016). As signals of more generalised (classed) competence, cultural capital signals may be valued differently in reference to different institutionalised human capital signals. With the importance of institutionalised qualifications for images of skill in Germany, cultural capital may, for instance, not interact with human capital as much. Then, the prototypically different skill formation systems may affect the interactions of human and cultural capital signals.

Moreover, in England, people are more prone to downplaying class identities than in Germany. While most people identify as working class in England; for Germany, the most common category of self‐identification is middle class (D’Hooge et al. 2018; Oesch and Vigna 2023). In England, this means that most people deflate their class identities. This identification with the working class, as well as the deflating, may reflect sensitivity towards class in the English case.

## 4 Methodology

### 4.1 Experimental Design

To better understand the interactions of cultural and human capital signals in the evaluation of job candidates, we conducted a factorial survey experiment with hiring agents. In factorial survey experiments, participants rate vignettes as short descriptive texts that experimentally vary on a range of factors (dimensions) (Auspurg and Hinz 2015). Because participants are required to weigh different factors simultaneously, and therefore against each other, the approach is fitting for studying judgements and evaluations. This method then allows us to examine the evaluations of hiring agents as gatekeeping in depth. Especially in comparison to traditional survey questions, the multidimensionality of evaluations that stems from using different treatments at the same time (different attributes) reduces the risks of social desirability biases. Moreover, the approach is well‐suited for studying interactions between signals that are typically highly correlated in real‐world settings. Considering the research question, cultural and human capital signals are usually correlated and some combinations, such as having a vocational training and an upper‐class background are rare.

The profiles that we presented to participants varied on seven vignette dimensions (1) cultural capital signals, (2) early/late career, (3) education level, (4) education specificity, (5) training, (6) working experience, and (7) gender (see Table 1 for additional information). We varied the early/late‐career dimension between respondents to avoid potentially overbearing age effects (Lössbroek et al. 2021). Respondents were either asked to evaluate (1) early‐career (around 25 years old) or (2) late‐career (around 55 years old) professionals to cover a large span of working lives. The six remaining dimensions were varied within respondents.

**TABLE 1:** Overview of vignette set‐up.

	Early‐career candidate	Late‐career candidate
Cultural capital	(1) Lower	(1) Lower
	(2) Higher	(2) Higher
Education level	(1) Vocational training (England: Higher apprenticeship, NVQ 3; Germany: Firm‐based vocational training)	(1) Vocational training (England: Higher apprenticeship, NVQ 3; Germany: Firm‐based vocational training)
	(2) University qualification (Bachelor level)	(2) University qualification (England: Bachelor level, Germany: Magister/Diplom)
Education specificity	(1) General field of study	(1) General field of study
	(2) Specific field of study that closely matches occupation	(2) Specific field of study that closely matches occupation
Training participation	(1) No indication	(1) No indication
	(2) Has participated in intensive, highly relevant training during the last year	(2) Has participated in intensive, highly relevant training during the last year
Work experience	(1) Work experience in adjacent fields & different firms for last 4 years	(1) Work experience in adjacent fields & different firms throughout career
	(2) Work experience in same profession & firm for last 4 years	(2) Work experience in same profession & firm throughout career
Gender	(1) Male	(1) Male
	(2) Female	(2) Female

Education Level was included as (1) vocational training or (2) university qualifications. We specified the names of universities to control for possible class‐based biases (i.e., higher cultural capital signals may be associated with prestigious universities). All included universities rank between the 100th and 200th best universities within the given field, and for England, we included Russell Group Universities (not Oxbridge or London‐based). We operationalised educational specificity as fields of study that closely match the given occupation, including relatively more general and specific qualifications relative to the specific sector. For instance, for the Software Developer job, Software Development and Computer Sciences are considered specific, and Engineering general fields of study. For training participation, vignettes either did or did not include information about this, as it would be unrealistic to indicate *not* having followed training. The training was specified as having taken place in the last year, as intensive and highly relevant. For work experience, we varied between (1) careers outside and (2) careers inside the field. We varied gender cues by using male‐ and female‐coded names and gendered pronouns.

### 4.2 The Cultural Capital Dimension

As described in the introduction, we chose to conceptualise and operationalise cultural capital by combining class backgrounds and current cultural practices, matching a Bourdieusian logic. We follow the idea that current practices are always marked by one's upbringing. Then, cultural capital signals necessarily connect the current class positions to people's class backgrounds (Bourdieu 1984; Lamont and Lareau 1988). Here, it is also important to stress that while evaluators may sometimes be more focused on social class backgrounds *or* practices in making sense of a person, they always make sense of the two together. With signals on class backgrounds, hiring agents will likely make assumptions about current practices, and vice versa.

The vignettes that we showed to hiring agents contained (1) more ordinary or (2) highbrow cultural capital signals (for an overview, see Table 2). To use cultural capital signals commonly available in hiring processes, we used first names and leisure activities. These were included in short descriptions of fictional candidates. While the classed connotations of leisure activities have long been established (Bourdieu 1984), prior research demonstrates similar tendencies for first names (see e.g., Crabtree et al. 2022; Elchardus and Siongers 2011; Gerhards 2010). We fixed these signal combinations: higher cultural capital profiles included upper‐class names and high‐status hobbies, while more ordinary ones featured more lower‐class names with more ordinary leisure activities. With this coupling of names and leisure activities, we operationalise the continuity between the names given by parents and leisure activities. More practically, we used this approach to create clear signals (combinations of names and leisure activities strengthen each other) and this coupling avoids weird scenarios that may draw additional attention (e.g., profiles with a lower‐class name and highbrow activities).

**TABLE 2:** Overview of cultural capital indicators used in vignettes.

	Lower cultural capital	Higher cultural capital
Leisure 1	Watch TV, to go to the gym with friends, and to listen to country (England)/schlager (Germany) music	Likes to play golf with friends, to go to the theatre, and to listen to classical music
Leisure 2	Likes to watch football with friends, to go for runs (England)/cycle (in Germany), and to play darts	Likes to sail, to play tennis, and to go to art museums
Names Germany	Justin, Kevin; Jacqueline, Chantal	Ferdinand, Konstantin; Charlotte, Elisabeth
Names England	Wayne, Kevin; Sharon, Chantelle	Rupert, Theodore; Arabella, Penelope

In our selection of signals, we aimed to establish clear, yet realistic profiles and to avoid systematic variation on other potentially relevant factors across the two levels (e.g., sociability, laziness, or fitness). We further conducted a pretest of different signals in Germany and England (see Supporting Information S1: Appendix Figures A1–A4). Based on this, we selected names and leisure activities that clearly signal high status and those rated lowest. A key goal was to harmonise the vignette design across the two countries. For the selection of names, we relied on the pretest, prior research on the stratification of names in Germany (Gerhards 2010) and mentions of discussions about names and social class in England (Jones 2020; Savage 2015).

To create clear signals for the leisure activities in the high cultural capital condition, we included traditional highbrow cultural activities. By selecting clear‐cut highbrow activities (such as visiting art galleries or the theatre), we set up this experiment as a more conservative, Bourdieusian test. The finally selected activities reflect the culturally‐ and economically dominant sides of the social space (Bourdieu 1984). With these choices, we follow prior research on the consequences of highbrow class signals (Reeves and De Vries 2019; Thomas 2018, 2022), rather than research on changing modes of elite distinction (Peterson and Kern 1996) or on more subtle, embodied forms of cultural capital (Van Der Waal et al. 2024).

For the counter scenario, we selected leisure activities that could be viewed as more ordinary, with some lower‐middle‐class connotations. We made this decision because selecting clear lower‐ or working‐class activities comes with a range of problems, including that working‐class consumption is largely characterised by a lack thereof (e.g., by lower sports participation for almost all sports), and by non‐distinctive activities such as gardening, board games, or arts and crafts activities (Bennett et al. 2009; Mutz and Müller 2021). For the purposes at hand, indications of a lack of cultural participation (e.g., not indicating sport) or only using home‐based hobbies (e.g., playing video games) would risk signalling lacking sociability, laziness, or unhealthy lifestyles.

For both the high and more ordinary cultural capital vignettes, we specified the leisure activities as combinations of sports and cultural consumption. To balance sociability across dimensions, we added ‘with friends’ to indicate sociability. By technically controlling for some social class stereotypes (such as for bodily fitness), we can ensure that these are not the reasons driving the differences in evaluations.

### 4.3 Sampling and Data

In this study, we only surveyed people with substantial and recent hiring experience within the specific fields. All participants of the factorial survey experiment had been involved in multiple hiring processes for positions in HR management or Software Development within the past 12 months prior to the experiment. This makes for a research population that is very narrow and not easy to reach.

We conducted the survey between May and September 2024. In Germany, we contacted hiring agents via email addresses listed in job ads for various positions in HRM and Software Development. Between April and June 2024, we searched for these advertisements via the German Governmental Unemployment Agency, which officially pools all job ads. We contacted around 1300 possible participants in each sector, including smaller and larger cities from each federal state. The approach resulted in 99 responses for HRM and 94 for Software Development.

This sampling strategy was not feasible for England, as most companies refrain from listing contact details in job ads. Through this approach, we reached fewer than ten respondents before we began sampling through LinkedIn. On the portal, we contacted recruiters, hiring agents, and software developers (who play a crucial role in hiring processes), approaching 2000 possible participants for Software Development and around 1500 for HRM. As this approach did not lead to the required response rate in HRM, we also recruited participants through Prolific, a UK‐based survey platform. As we could restrict participation to those who work in HRM and who have hiring experience, we considered this a viable strategy to reach professionals with hiring experience in the sector. We emphasised that prior hiring experience was a prerequisite and included two screening questions in the survey. We included an attention test. The different strategies resulted in 96 responses in HRM (61 from Prolific) and 75 in Software development (see Supporting Information S1: Appendix Tables A1 and A2 for respondent and firm characteristics). We will address our response rates and different sampling strategies in the discussion.

All respondents evaluated eight profiles for (a) HR Manager or (b) Software Development positions. It is important to note that for factorial survey experiments, the unit of analysis the vignette rather than the respondent level. What we are interested in are, in the end, the evaluations of candidates. Hence, the sample size at the vignette level is crucial (*N* respondents * 8 vignettes). Auspurg and Hinz (2015, 47) suggest that all vignettes should be evaluated by several respondents. Specifically, their recommendation, even to be able to identify the effects of respondents' variables (which we do not do) is ‘allocating at least five different respondents to each vignette or deck’. This condition is fulfilled across the contexts.

The different combinations of the dimensions make for a total of 64 vignettes ($2^{6}$), for both the early and late‐career cases. We used the state‐of‐the‐art D‐efficient design to block the vignettes into decks (Auspurg and Hinz 2015), where eight blocks of eight vignettes each made for a highly efficient set‐up. D‐effecient designs maximise statistical power, in part by ensuring that most levels of each dimension are evaluated by each respondent. In our solution, all vignette levels appear equally often in each vignette (i.e., every respondent evaluates four vignettes with lower and for with higher cultural capital signals, four with vocational and four with university education, etc.). Moreover, the design ensures that all main dimensions and two‐way interactions are uncorrelated (orthogonal). We randomised (1) whether respondents were assigned to the early or late‐career condition, (2) which blocks they rated and (3) the order of vignettes within blocks.

For the outcome variable, participants were asked to judge the likeliness of hiring candidates on a scale from 0 to 10. We preferred this question to the likeliness to invite candidates for interviews as the likeliness to employ is more consequential. For respondents who evaluated early or late‐career vignettes, we did not change the wording regarding the seniority in the position, meaning that respondents in the early and late‐career condition responded to the same question as the dependent variable (see Supporting Information S1: Appendix Figure A5 for an example).

### 4.4 Analyses

As is common in factorial survey research, we use random intercept models to account for the nested structure of the data (Auspurg and Hinz 2015). All models predict the likeliness to hire the candidate as the dependent variable. The independent variables include the vignette dimensions, and we always control for the evaluated vignette set.

To understand the main effects, we first run separate country models with dummy variables for the sector and career stage to control for contextual variation. Then, we further separate the model specification by sectors (including the career control) (Models 2 and 3, see Supporting Information S1: Appendix Tables A3 and A4) and next by the early/late‐career condition (including the sector dummy) (Models 4 and 5, see Supporting Information S1: Appendix Tables A3 and A4).

We then include interaction terms between the cultural capital dummy and (1) education level, (2) education specificity, (3) training, and (4) work experience to examine the interactions between human and cultural capital signals (Models 6–10, Table 3). We first run these models separately by country (controlling for career stage), then by career stage and sector. Before interpreting individual interactions, we first conduct a joint Wald test to examine whether the four interactions between the human and cultural capital signals are *jointly* different from zero (see Table 4 for an overview of all joint Wald Tests). In other words, this test informs us whether the four interactions jointly contribute to the model (Veazie 2006). We do so as, based on our theory, we do not have a clear reason to expect that each of the four human capital indicators will moderate the cultural capital signal in a different way. Instead, the four interactions *all* relate to the *same* theoretical hypothesis that there *is* an interaction between cultural and human capital signals. The large number of significance tests required to test all interactions separately would increase the chance of type 1 errors, of wrongly finding statistically significant results. To reduce this risk, we will only consider the separate interaction effects in case our joint Wald test is statistically significant. Inspecting the individual interactions will allow us to shed more light on whether and how *specific* human capital indicators moderate cultural capital signals. To get further insight into the dynamics of the evaluations, we calculate the marginal effects of cultural capital dependent on the human capital variables.

**TABLE 3:** Interactions by sector and career stage (models 6–10).

	Germany					England				
	M6a: Combined	M7a: HRM	M8a: Software	M9a: Early career	M10a: Late career	M6b: Combined	M7b: HRM	M8b: Software	M9b: Early career	M10b: Late career
Cultural capital	−0.31^*^	−0.20	−0.38^*^	−0.02	−0.61^**^	−0.14	−0.32	0.10	−0.24	−0.02
(Ref. lower)	[0.14]	[0.21]	[0.19]	[0.19]	[0.20]	[0.16]	[0.22]	[0.24]	[0.25]	[0.21]
Education level	0.24^*^	0.19	0.31	0.34^**^	0.14	0.71^***^	0.72^***^	0.71^***^	0.73^***^	0.70^***^
(Ref. vocational)	[0.10]	[0.12]	[0.16]	[0.13]	[0.15]	[0.12]	[0.18]	[0.16]	[0.16]	[0.18]
CC * level	0.26^*^	0.29	0.21	0.13	0.40^*^	−0.21	−0.28	−0.16	−0.24	−0.20
	[0.13]	[0.17]	[0.19]	[0.18]	[0.18]	[0.16]	[0.22]	[0.24]	[0.23]	[0.23]
Education specificity	0.98^***^	0.87^***^	1.11^***^	1.01^***^	0.96^***^	0.67^***^	0.75^***^	0.56^***^	0.43^**^	0.91^***^
(Ref. general)	[0.09]	[0.12]	[0.14]	[0.13]	[0.14]	[0.11]	[0.16]	[0.16]	[0.15]	[0.16]
CC * specificity	0.21	0.11	0.29	0.23	0.19	0.01	0.11	−0.11	0.27	−0.26
	[0.12]	[0.16]	[0.17]	[0.17]	[0.17]	[0.14]	[0.20]	[0.22]	[0.18]	[0.22]
Training	0.44^***^	0.29^*^	0.62^***^	0.56^***^	0.33^**^	0.67^***^	0.50^**^	0.89^***^	0.61^***^	0.75^***^
	[0.09]	[0.11]	[0.15]	[0.14]	[0.13]	[0.12]	[0.18]	[0.16]	[0.17]	[0.17]
CC * training	0.23	0.34^*^	0.08	−0.06	0.53^**^	0.15	0.29	−0.05	0.19	0.09
	[0.13]	[0.15]	[0.21]	[0.19]	[0.17]	[0.15]	[0.21]	[0.20]	[0.21]	[0.22]
Work experience	2.41^***^	2.15^***^	2.68^***^	2.84^***^	1.95^***^	2.30^***^	2.39^***^	2.19^***^	2.40^***^	2.20^***^
	[0.18]	[0.23]	[0.28]	[0.23]	[0.28]	[0.18]	[0.22]	[0.30]	[0.24]	[0.27]
CC * experience	0.22^*^	0.16	0.28	0.04	0.41^**^	0.26^*^	0.39^*^	0.09	0.36^*^	0.15
	[0.10]	[0.13]	[0.16]	[0.14]	[0.15]	[0.13]	[0.19]	[0.15]	[0.18]	[0.18]
*N* vignettes	1544	792	752	792	752	1368	768	600	696	672
*N* respondents	193	99	94	99	94	171	96	75	87	84

*Note:* Stated Likeliness of Hiring the Candidate (0–10). Regression models of interaction effects between human and cultural capital signals across contexts. Only containing relevant effects, see Supporting Information S1: Appendix Tables A5 and A6 for full specifications. Standard Errors are shown in brackets.

^*^ *p* < 0.05.

^**^ *p* < 0.01.

^***^ *p* < 0.001.

**TABLE 4:** Overview of joint tests (Wald tests).

Context	Model	Germany			England		
		Chi^2^ (4)	*p*‐value	*N* vignettes	Chi^2^ (4)	*p*‐value	*N* vignettes
Combined contexts	6	13.68	0.008*	1544	6.47	0.167	1368
Early career	9	9.02	0.061	792	7.85	0.097	768
Late career	10	7.53	0.110	752	1.17	0.884	600
HRM	7	2.30	0.680	792	6.77	0.148	696
Software development	8	18.53	0.001*	752	2.97	0.562	672

*Note:* Wald tests as joint hypotheses tests of the different interaction effects, testing against the Null Hypothesis that there are no interactions between human and cultural capital signals. The test then is: H0: Cultural Capital × Education Level = Cultural Capital × Work Experience = Cultural Capital × Specificity = Cultural Capital × Training = 0. The tests refer to Models 6–10 (Table 3, Supporting Information S1: Appendix Tables A5 and A6).

## 5 Results

### 5.1 Descriptive Statistics

Table 5 displays the descriptive statistics of the dependent variable across the different contexts. The means of the evaluated likeliness to hire candidates on the scale from 0 to 10 varied between 5.4 and 6.2 points. On average, late‐career profiles in Germany and candidates in the German Software Development sector were rated the lowest, and the highest ratings were in England in Software Development. The standard deviations are similar across contexts, ranging between 2.6 and 2.7 points. Note that we do not discuss the averages of the other variables that we use in the model (the dimensions of the vignettes) because they all are equal to 0.5, as half of the vignettes are coded as 0 and the other as 1.

**TABLE 5:** Descriptive statistics for the dependent variable.

	Germany				England			
	Mean	SD	*N* vignettes	*N* respondents	Mean	SD	*N* vignettes	*N* respondents
Combined	5.6	2.7	1544	193	5.8	2.6	1368	171
HRM	5.9	2.6	792	99	5.5	2.7	768	96
Software development	5.4	2.8	752	94	6.2	2.6	600	75
Early career	5.9	2.7	792	99	6.0	2.7	696	87
Late career	5.4	2.7	752	94	5.7	2.6	672	84

### 5.2 Main Effects

**FIGURE 1:**
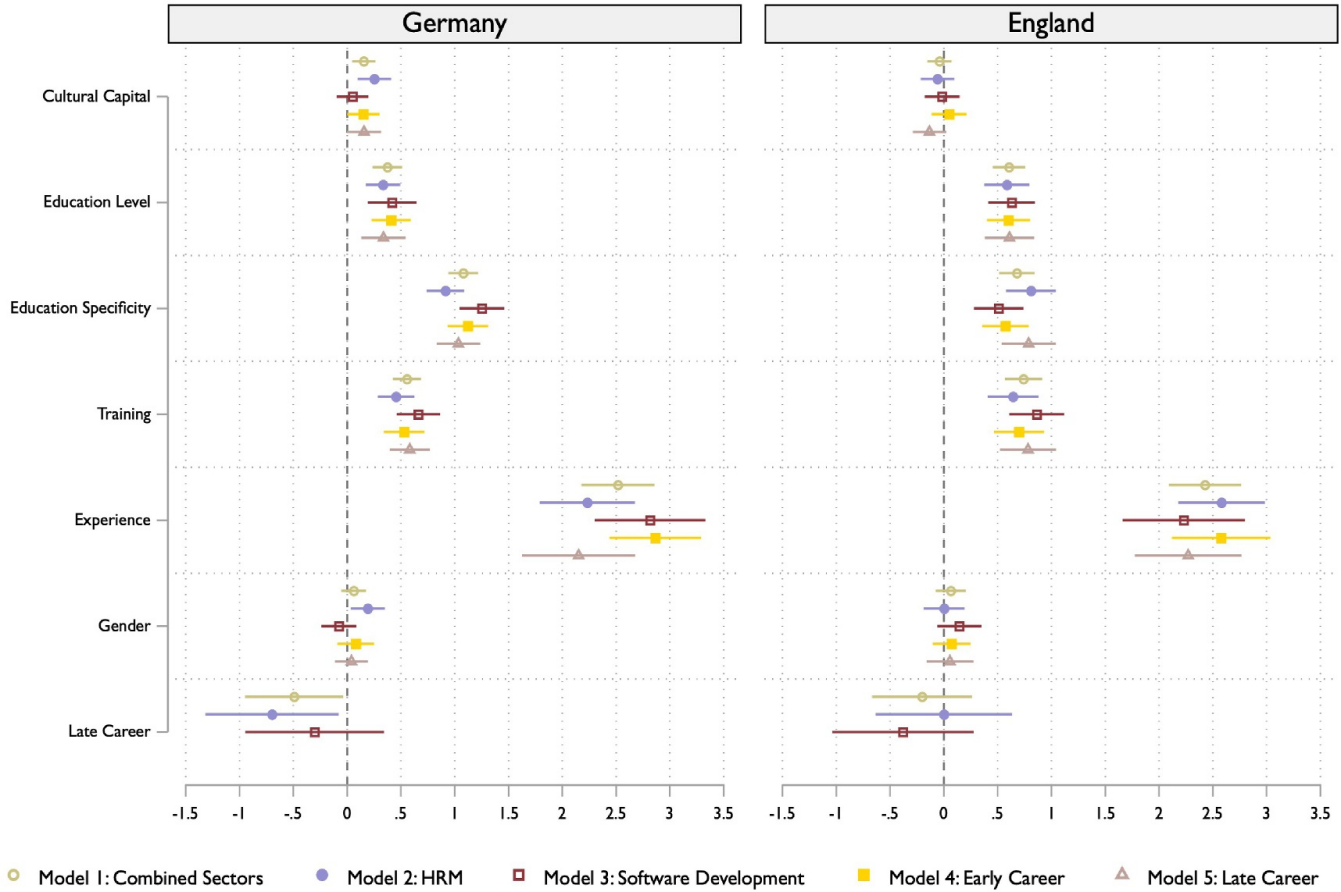
Main effects for the vignette dimensions by country, sector and career stage. This Figure is based on Model 1–5, see Supporting Information S1: Table A3 for Germany and Table A4 for England. *N* vignettes Germany = 1544, *N* vignettes Germany HRM = 792, *N* vignettes Germany software = 752, *N* vignettes Germany early = 792, *N* vignettes Germany late = 752. *N* vignettes England = 1368, *N* vignettes England HRM = 768, *N* vignettes England software = 600, *N* vignettes England Early = 696, *N* vignettes England late = 672. The whiskers denote 95% confidence intervals.

Figure 1 displays the findings of Models 1 to 5 (see Supporting Information S1: Appendix Tables A3 and A4), focused on the main effects. First, we combine both sectors and career stages (Model 1), then run separate sector models (for HRM software development), and separate early/late career models. As visible when inspecting the confidence intervals in Figure 1, cues of cultural capital did not confer advantages in many contexts.

In England, high cultural capital signals do not confer advantages in either field. On the eleven‐point scale, in HR, the effect is −0.06 and in Software Development −0.02, two null effects. Similarly, the effects of cultural capital for the early and late career are not significantly different from zero when we combine the sectors.

In Germany, on average, high cultural capital signals paid off, for evaluations of early and late‐career applicants. When comparing sectors, we find a null effect for the Software Development case and a relatively pronounced effect in the HRM sector. The HRM hiring agents rated high cultural capital candidates 0.25 points (on the scale from 0–10) higher on average than candidates with more ordinary cultural capital signals. The average rating of candidates with lower cultural capital indicators was 5.78 and for those with higher cultural capital indicators 6.04. While the effect is relatively small in relation to most human capital indicators, the effect size is not statistically different from the benefit of university qualifications over vocational training (0.33).

As visible in the effects shown below the cultural capital effects in Figure 1, hiring agents give value to all human capital signals across the contexts. Prior work experience in the field is valued particularly, and in the German case, the specificity of education is valued highly. For the indications of candidates' gender, we only find a positive effect in HRM in Germany.

### 5.3 Interactions Between Cultural and Human Capital Signals

**FIGURE 2:**
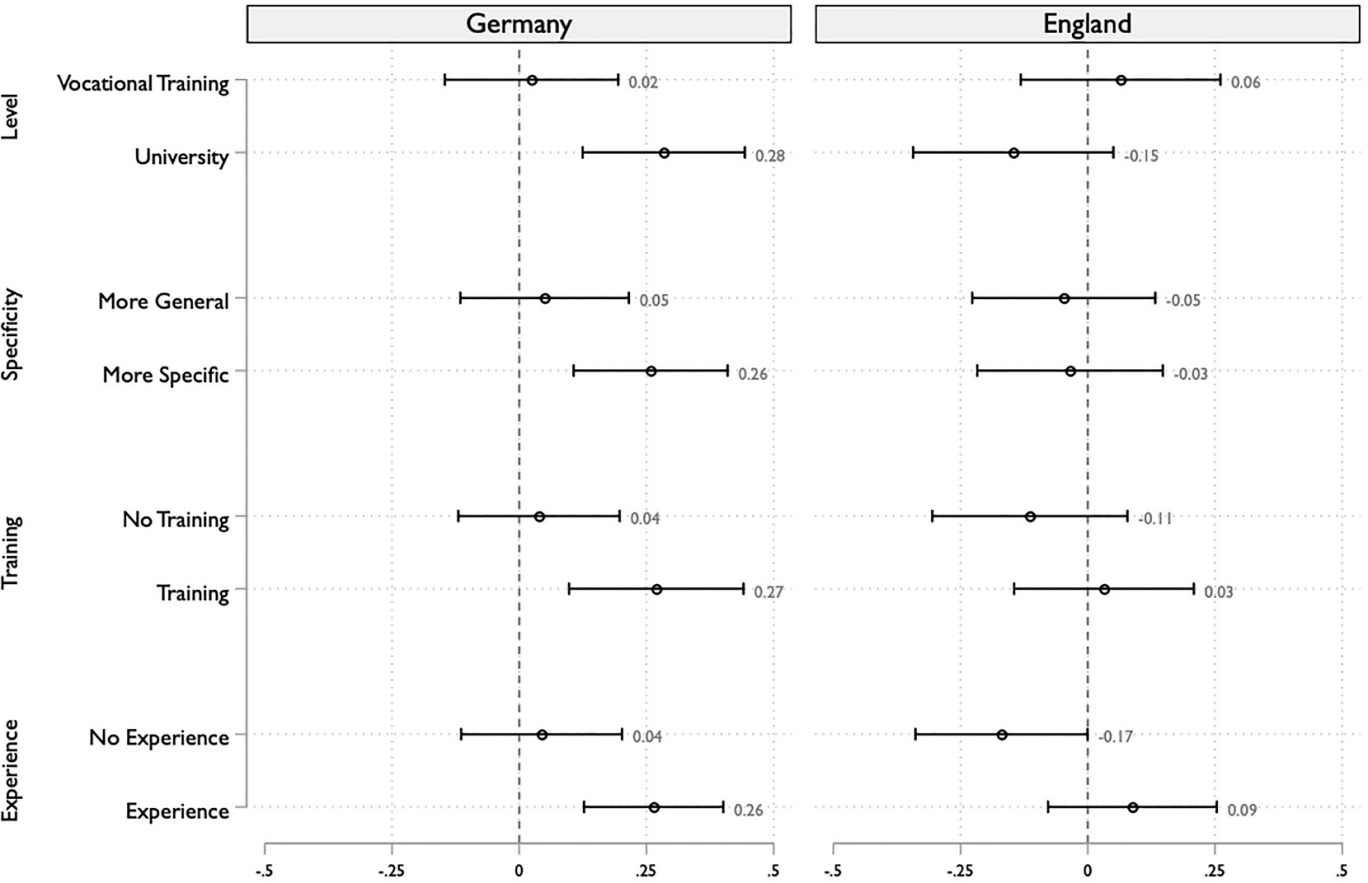
Marginal effects of cultural capital signals as dependent on human capital signals across countries. This figure is based on separate country models that include all interaction terms next to the main dimensions, see Table 3, Model 6a and 6b; and Supporting Information S1: Appendix Tables A5 and A6. We plot the marginal effect of cultural capital signals on the dependent variable at different levels of human capital. *N* vignettes Germany = 1544, *N* vignettes England = 1368. The whiskers denote 95% confidence intervals.

**FIGURE 3:**
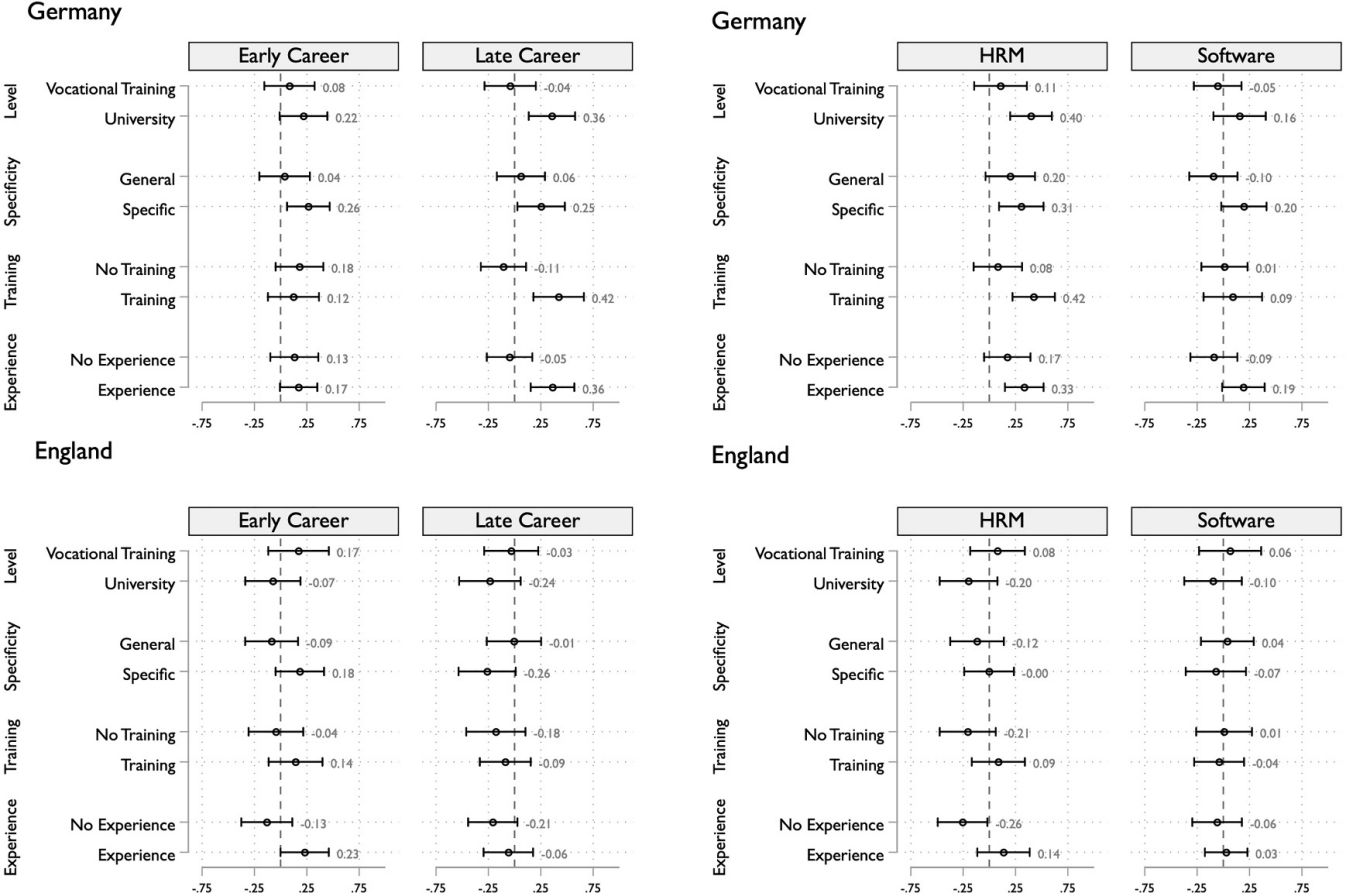
Marginal effects of cultural capital signals as dependent on human capital signals across countries, by career and sector. This Figure is based on separate country, sector and career models that include all the interaction terms next to the main dimensions (Models 7–10, Table 3, Supporting Information S1: Appendix Tables A5 and A6). We plot the marginal effect of cultural capital signals on the dependent variable at different levels of human capital. *N* vignettes Germany early career = 792, *N* vignettes Germany late career = 752. *N* vignettes Germany HRM = 792, *N* vignettes Germany software = 752; *N* Vignettes England Early Career = 696, *N* vignettes England late career = 672. *N* Vignettes England HRM = 768, *N* vignettes England software = 600. The whiskers denote 95% confidence intervals.

Next, we turn to the interactions between cultural and human capital signals (see Table 3 for an overview). As visualised in Figure 2, these interactions look different across the two countries. Here, Figure 2 (like Figure 3) displays the marginal effects of cultural capital signals dependent on human capital signals. For instance, the first confidence interval displays the average value of cultural capital signals for candidates with vocational training, and the second one displays that for those with university qualifications.

Starting with Germany, the joint hypothesis test (see Table 4) provides evidence for interactions between the cultural and human capital signals (*p* = 0.008). This surfaces as a consistent multiplication pattern: high cultural capital only paid off when candidates also signalled high levels of human capital, not for those with lower human capital signals. Although not all individual interaction effects are statistically significant, their similar size and direction can be read as support for the finding. The interactions between cultural capital signals and (1) level of education (*p* = 0.041) and (2) working experience (*p* = 0.032) are statistically significant, while those with education specificity (*p* = 0.078) and training (*p* = 0.070) are slightly larger than the conventional alpha level of *p* ≤ 0.05. The effect sizes range between 0.21 (for specificity) and 0.26 (for the level of education) on the 11‐point scale.

Zooming in on the significant interactions, we see that cultural capital signals were not considered for those with vocational training. Here, candidates with vocational training were rated with 5.44 points on average if they had lower cultural capital signals, and with 5.47 points with higher cultural capital signals. For those with university qualifications, the average rating of candidates with lower cultural capital signals was 5.68 and for those with higher cultural capital signals, 5.97. Hence, those with university qualifications experience an average cultural capital benefit of 0.3 points. This pattern looks similar for the interaction with work experience: candidates without prior work experience in the relevant field did not benefit from cultural capital signals (0.02), while those with a prior career in the field benefitted, on average, by 0.28 points from cultural capital signals.

For England, there is no support for the joint test; overall, we cannot conclude that evaluations of cultural and human capital signals were affected by each other (*p* = 0.167). Upon closer inspection, we note that the interaction between cultural capital and work experience points to a statistically significant effect (*b* = 0.26*; *p* = 0.04). There is a slight preference for lower cultural capital signals in the absence of work experience and a null effect for candidates *with* field‐specific work experience.

### 5.4 Interactions Across Contexts

Sectors and career stages further shape the interactions. Figure 3 depicts the value of cultural capital as dependent on human capital across the two sectors, as well as in the early and late career for Germany (above) and England (below). For both countries and the four models (by career and sector), we run Wald tests to jointly test the interactions between the human and cultural capital signals (see Table 4).

In Germany, the Wald test provides evidence for a multiplication effect in the late career: evaluations of cultural capital are affected by human capital signals. Considering individual interactions for late‐career candidates, evaluations of cultural capital signals were affected by training, level, and work experience. Here, high cultural capital was only valuable in combination with higher human capital indicators. These interaction effects are substantial: candidates with university education benefit an average of 0.40 points more from cultural capital signals than those with vocational training; for those with training it is 0.53 points compared to those without training, and for those with work experience the advantage is 0.41 points on average. For early‐career candidates in Germany, the Wald test was not statistically significant, suggesting that cultural capital evaluations were not affected by human capital signals. When looking at the two sectors separately, the Wald test is not statistically significant in either sector. When we zoom in on HRM, it is evident that the Wald test is just below the significance level, with *p* = 0.06. Considering individual interactions, only the interaction between cultural capital signals and training is statistically significant, suggesting a multiplication pattern. For Software Development, the Wald test and all individual interactions are not statistically significant.

There are no joint interaction effects across the four contexts in England. This is reflected in almost all individual interactions not being statistically significant. Hence, evaluations of cultural and human capital were unaffected by each other. The only statistically significant interaction term that we find, in the early career and in HRM, is a multiplication effect between work experience and cultural capital. We note with caution that, in some instances, hiring agents in England seemed to prefer lower cultural capital profiles to higher ones. Considering that the joint test and individual interactions do not reach statistical significance, this should be read as a descriptive pattern.

## 6 Discussion and Conclusion

Cultural capital signals are not a universal labour market currency, but can be contingent on human capital signals in the way they inform hiring decisions. To better understand potential links between human and cultural capital signals in labour market allocation; specifically, about how the two types of signals interact in evaluations, we conducted a factorial survey experiment with hiring agents in Software Development and HRM. Based on the results presented above, to the question of how hiring agents evaluate signals of (highbrow) cultural capital, considering different human capital signals, is two‐fold: (1) in the contexts we studied, hiring agents often do not draw on these types of signals and (2) human capital signals often serve as a necessary condition for cultural capital signals to be valued.

In the fields we studied, the value of highbrow cues was especially limited in the English context. For software development and HR positions, hiring agents in England hardly gave value to cultural capital signals, irrespective of displayed human capital signals. In Germany, their value was contingent on each other. While the evaluations of cultural capital signals were consistent in the early career, outside of this context, we found evidence for multiplication patterns. Hiring agents then valued cultural capital signals when they were combined with human capital indicators. The multiplication pattern was especially prevalent for late‐career candidates in Germany. In the late career, the observed interdependencies pertained to training, work experience, and the level of education. This could stem from late‐career worker ideals and expectations about ‘expanded roles’ (Friedman and Laurison 2020; Meyer 1977).

These findings have theoretical implications for the labour market value of (highbrow) cultural capital specifically and the reproduction of inequalities more generally. On the demand side, the benefits of such signals do not necessarily derive from additive effects, but hiring agents evaluate candidates particularly positively when they combine cultural and human capital signals. Beyond increasing value through combinations, however, the value of cultural capital signals may need to be activated in the first place. It is partially conditional on human capital, which matches the findings of Reeves and De Vries (2019) that cultural consumption does not generate benefits for everyone but that this process depends on university qualifications. In combination, these findings raise questions about the necessary conditions for highbrow cues to generate labour market value. This applies not only to institutional contexts (e.g., countries and professions) and to individual characteristics (e.g., age or career stage), but also to the situational problems that hiring agents address through evaluating on class signals. The results suggest that they serve as a differentiator between skilled candidates (Rivera 2015), rather than to make decisions under uncertainty (Fossati et al. 2020).

Further, we find no evidence for other interaction patterns; not for compensation or the (de‐)valuation of mobility patterns. This is also relevant as the judgements of mobility patterns could counter the reproduction of inequalities. Here, it is important to note that not finding clear patterns may also result from different mechanisms (e.g., compensation and the reward of upward mobility) being applied simultaneously and offsetting one another.

A key decision we made in setting up the study is the conceptualisation of cultural capital as the fixed combination of cultural practices with class backgrounds through names. While we have described the reasons why we made the choice throughout the paper, a crucial shortcoming is that we cannot disentangle the signalling of class backgrounds from current classed practices. Signalling someone's class background more explicitly, potentially by including remarks about being a first‐generation student, could have foregrounded mechanisms around social mobility. Future quantitative studies, as well as qualitative research using interview‐elicitation methods, could further disentangle the relationship between inherited class markers and acquired cultural signals in combination with human capital signals.

Next, a potential concern are socially desirable answering patterns (e.g., Pager and Quillian (2005) whose publication focuses on ethnic discrimination, which is especially prone to social desirability). This may be favoured by our decision to use strong social class indicators. In Germany, we potentially underestimate the effect; however, the hiring agents' use of non‐meritocratic criteria points against social desirability. For England, the evaluation patterns more closely follow socially desirable expectations. Here, it is important to note our approach of pretesting, as well as the strength of the factorial survey approach in not evoking bias. Moreover, if this type of bias shapes the results in England, we want to underline that these moral concerns, too, are sociologically meaningful. They may matter as hiring agents justify their work and decisions to themselves (De Keere 2023) and others; and are a relevant social fact in and of themselves (Lamont and Swidler 2014).

Another limitation is the sampling process. We attribute our low response rates to the specificity of our (hard to reach) research population, the length of the survey, the voluntary set‐up without financial compensation that hiring agents were emailed with; as well as the overflow of LinkedIn requests in the software and HRM field. We do not see reason to expect strong biases in non‐response and believe that the voluntary targeting of this narrow population also makes for high‐quality answers. For country differences in sampling approaches, we especially want to point to the use of Prolific in England. We did not find significant differences between the HR prolific respondents and the remaining HR respondents on various indicators (see Supporting Information S1: Appendix Table A7). Still, some patterns raise the possibility of slight status differences, which may also affect the results.

Finally, particularly when introducing interaction effects, we may lack the statistical power to identify smaller effects. This means that smaller individual effects (e.g., the interaction between the level of education and cultural capital in HRM in England) may indeed be underpowered and may have been statistically significant with a larger sample size. Here, it is important to note that in Germany, we found significant main effects that can then be explained through advantages for candidates with high cultural capital. We consistently saw significant marginal effects for the combined analyses. Moreover, especially for the late career condition, the effects are evident. In England, this is not the case: there were no significant main effects to begin with, and most margins are not significant. Hence, in the results there hardly were instances where we could say that cultural capital signals matter for England. We note individual significant interactions, but these are not sufficient to conclude that cultural capital signals are then dependent on human capital signals. Moreover, we followed the sample size recommendations with at least five respondents per set and used the d‐efficient approach to increase power. Finally, the more conservative approach of jointly testing hypotheses means that we can be sure of what we *do* find (with inevitable risks for type 2 errors).

Despite these limitations, the results of our study match prior research that shows how cultural capital signals do not function as a universal structuring mechanism in all professional contexts. While prior research has clearly demonstrated that signals of cultural capital—and social class more broadly—play a role in hiring and career progression in elite, professional service firms in the UK (e.g., Friedman and Laurison 2020), there may be professional contexts where hiring agents are not as prone to distinguish based on such cues. This sector‐specific pattern supports Erickson's (1996) earlier claim that upper‐class signals are not uniformly useful for creating and maintaining workplace distinctions and matches Thomas' (2018) and Streib's (2023) results. This context‐dependency does not only apply to the net value of the signals, but also to their dependency on human capital signals. Hiring agents' ways of evaluating cultural capital signals are situation‐, context‐ and field‐dependent.

The factorial survey approach also gives several advantages that we could draw upon in this paper in contributing to the current literature. First, with this approach, we zoomed in on hiring evaluations as a setting where combinations of signals can create advantages. Beyond observing patterns of labour market trajectories, we can examine the moment and mechanism of evaluation. Second, the factorial survey approach allowed us to study similar vignettes, similar cultural and human capital signals in different contexts. Third, with the approach we could disentangle cultural from human capital signals, which would be highly correlated in survey data. We could, for instance, create scenarios of candidates who have done vocational training and yet consume high‐brow culture. While we then make sacrifices regarding external validity (for instance, when compared to an audit study where we would send out applications), this survey set‐up offers flexibility in presenting the vignettes, allowing us to tease out context‐specific interactions within evaluations.

As we conducted the experiment with hiring agents located in the specific fields of software development and HRM, we cannot generalize our results to other cases. We do believe, however, that specificity of professional fields in shaping the currency of cultural capital signals is not only theoretically but also methodologically important. First, to account for the context dependency of professions, surveys should target participants who make hiring decisions within these specific fields. When targeting samples of hiring agents who hire across fields or when targeting general population samples, we ignore the context of the evaluations that are crucial to tracing inequality‐generating mechanisms. Second, the country variations call for comparative research on cultural capital evaluations in hiring processes in the future. Due to the strong opportunities for comparisons, survey experiments could be a valuable approach here. Here, the value of cultural capital signals in relation to human capital signals in blue‐collar professions, or with an emphasis on class backgrounds could be important pathways.

To conclude, we emphasise the importance of examining the interplay between different sets of signals to understand how evaluations come about in specific settings. These signals also interact in the evaluative practices of hiring agents: human capital appears to function as a necessary condition, while the value of cultural capital arises through particular combinations. Even though these two concepts are rooted in distinct theoretical traditions, a sociology of evaluation perspective sensitizes us to their interaction and offers an important pathway for understanding the labour‐market value of cultural‐capital signals beyond elite professions.

## Funding

This work was supported by the European Research Council starting grant [Grant Nos. ID: 950189 to TB].

## Ethics Statement

The AISSR Ethics Advisory Board from the University of Amsterdam approved the data collection for the project in February 2022.

## Conflicts of Interest

The authors declare no conflicts of interest.

## Supporting information


Supporting Information S1

## Data Availability

The data will be made publicly available at the end of the project duration. Before that, they are available from the corresponding author on reasonable request.

## Endnotes

^1^
We refer to human capital signals as specific and more general work‐related skills or qualifications that could colloquially be referred to as human capital. We do *not* adopt theoretical assumptions from human capital theory about rationality in the acquisition and evaluation of capital (Becker 1962).

^2^
Prior research that points to such patterns is mostly not based on employer samples. Instead, it includes research on the wider population (Reeves and Friedman 2024) and people with lower social class positions (Jarness and Flemmen 2019). These groups may have more critical stances towards high cultural capital profiles as well as different interests and goals.

^3^
In 2024, in Germany, 85.8% of professionals in Software Development identified as men (Statistik der Bundesagentur für Arbeit 2025) and in the UK, 80% identified as men (Techtalent Charter 2024).

^4^
In Germany, in 2024, 73.6% of HR professionals in Germany identified as women (Statistik der Bundesagentur für Arbeit 2025). In the UK, 87.5% of professionals in HR administrative occupations, 67.7% of HR and IR officers and 60.9% of HR Managers identified as women in 2021 (Ainsworth and Pekarek 2022).
