# Supplementary material for: Cultural and Human Capital Signals in Hiring—A Factorial Survey Experiment Across Contexts
Source: Br J Sociol. 2026 May 20;77(4):786–801. doi: 10.1111/1468-4446.70125 (PMC13536517; doi:10.1111/1468-4446.70125)
Supplement: Supplementary file 1 [file BJOS-77-786-s001.docx]

**Table A1**: Respondent and Firm Information by Sector, Germany

Variable

HRM

Software

|  | Mean | Median Std | | N | Mean | Median Std | | N |
| --- | --- | --- | --- | --- | --- | --- | --- | --- |
| Firm Information Firm Size (employees) | 1188 | 235 | 3394 | 74 | 826 | 105 | 2810 | 72 |
| Base Income (€) | 45155 | 45000 | 7955 | 94 | 52089 | 50000 | 10765 | 93 |
| Firm Higher Education (0–3  scale) | 1.2 |  |  | 143 | 1.8 |  |  | 149 |
| Age Distribution *<*35 years | 33% | 0.28 | 0.19 | 74 | 39% | 0.30 | 0.18 | 74 |
| 35-55 years | 45% | 0.45 | 0.15 | 74 | 43% | 0.44 | 0.13 | 74 |
| *>*55 years | 21% | 0.20 | 0.17 | 74 | 18% | 0.19 | 0.13 | 74 |
| Respondent Information  Gender (%  Women/Non-Binary) | 83% |  |  | 96 | 72% |  |  | 93 |
| Age (years) | 42.0 | 42.0 | 12.5 | 95 | 40.0 | 38.0 | 10.8 | 93 |
| External Hiring (% External) | 22% |  |  | 99 | 21% |  |  | 94 |
| Education (% University) | 68% |  |  | 90 | 70% |  |  | 88 |

*Notes:* Firm-level and respondent-level descriptive statistics for Germany, by sector (HRM and Software). Higher education (0–3 scale) represents the share of employees with tertiary education within the firm.

### **Table A.2:** Respondent and Firm Information by Sector, England

Variable

HRM

Software

|  | Mean | Median Std | | N | Mean | Median Std | | N |
| --- | --- | --- | --- | --- | --- | --- | --- | --- |
| Firm Information Firm Size (employees) | 3063 | 400 | 8532 | 75 | 16177 | 250 | 66668 | 56 |
| Base Income (£) | 42271 | 41000 | 11423 | 96 | 34460 | 32000 | 9174 | 75 |
| Firm Higher Education (0–3  scale) | 1.5 |  |  | 94 | 2.2 |  |  | 55 |
| Age Distribution *<*35 years | 37% | 0.30 | 0.20 | 75 | 44% | 0.49 | 0.24 | 57 |
| 35-55 years | 43% | 0.43 | 0.15 | 75 | 40% | 0.35 | 0.20 | 57 |
| *>*55 years | 21% | 0.19 | 0.16 | 75 | 16% | 0.10 | 0.14 | 57 |
| Respondent Information  Gender (%  Women/Non-Binary) | 70% |  |  | 70 | 36% |  |  | 73 |
| Age (years) | 41.0 | 39.0 | 9.7 | 69 | 35.0 | 33.0 | 8.3 | 69 |
| External Hiring (% External) | 22% |  |  | 96 | 21% |  |  | 75 |
| Education (% University) | 79% |  |  | 94 | 86% |  |  | 70 |

*Notes:* Firm-level and respondent-level descriptive statistics for England, by sector (HRM and Software). Higher education (0–3 scale) represents the share of employees with tertiary education within the firm.

**Figure A.1**: Pre-test First Names, Germany

*Source:* Pre-test Class Signals (Own Survey via Prolific, Own Figure). *Note*: Mean Ratings on a Status Ladder (0-10). *𝑁*_Respondents_ = 151.

**Figure A.2:** Pre-test Leisure Activities, Germany

*Source:* Pre-test Class Signals (Own Survey via Prolific, Own Figure).

*Note*: Mean Ratings on a Status Ladder (0-10). *𝑁*_Respondents_ = 151.

**Figure A.3**: Pre-test First Names, England

*Source:* Pre-test Class Signals (Own Survey via Prolific, Own Figure). *Note*: Mean Ratings on a Status Ladder (0-10). *𝑁*_Respondents_ = 150.

**Figure A.4**: Pre-test Leisure Activities, England

*Source:* Pre-test Class Signals (Own Survey via Prolific, Own Figure).

*Note*: Mean Ratings on a Status Ladder (0-10). *𝑁*_Respondents_ = 150.

**Figure A.5**: Example Vignette

*Source:* Qualtrics Survey

### **Table A.3**: Models with Main Effects, Germany

|  | M1a: Combined | M2a: HRM | M3a: Software | 4a: Early Career | M5a: Late Career |
| --- | --- | --- | --- | --- | --- |
| Cultural Capital (ref. lower) | 0.15^**^ [0.01] | 0.25^**^ [0.00] | 0.05 [0.50] | 0.15^*^ [0.05] | 0.16 [0.05] |
| Education Level (ref. Vocational) | 0.37^***^ [0.00] | 0.33^***^ [0.00] | 0.42^***^ [0.00] | 0.41^***^ [0.00] | 0.34^**^ [0.00] |
| Education Specificity (ref. General) | 1.08^***^ [0.00] | 0.91^***^ [0.00] | 1.25^***^ [0.00] | 1.12^***^ [0.00] | 1.03^***^ [0.00] |
| Training | 0.56^***^ [0.00] | 0.45^***^ [0.00] | 0.66^***^ [0.00] | 0.53^***^ [0.00] | 0.58^***^ [0.00] |
| Work Experience in Field | 2.52^***^ [0.00] | 2.23^***^ [0.00] | 2.82^***^ [0.00] | 2.86^***^ [0.00] | 2.15^***^ [0.00] |
| Gender (ref. Men) | 0.06 [0.30] | 0.19^*^ [0.02] | -0.08 [0.35] | 0.08 [0.35] | 0.04 [0.61] |
| Early/Late Career (ref. Early) | -0.49^*^ [0.03] | -0.70^*^ [0.03] | -0.30 [0.36] |  |  |
| Software (ref. HRM) | -0.58^*^ [0.01] |  |  | -0.79^*^ [0.01] | -0.40 [0.22] |
| Vignetteset 2 (ref. 1) | -0.47 [0.24] | -0.43 [0.38] | -0.40 [0.47] | 0.04 [0.95] | -1.02^*^ [0.02] |
| Vignetteset 3 (ref. 1) | -0.87^*^ [0.03] | -1.53^**^[0.00] | -0.26 [0.65] | -0.44 [0.45] | -1.36^**^ [0.01] |
| Vignetteset 4 (ref. 1) | -0.78 [0.13] | -1.08 [0.13] | -0.51 [0.50] | 0.09 [0.87] | -1.69^*^ [0.05] |
| Vignetteset 5 (ref. 1) | -0.93^*^ [0.02] | -1.67^**^ [0.00] | -0.13 [0.82] | -0.63 [0.32] | -1.30^*^ [0.01] |
| Vignetteset 6 (ref. 1) | -1.10^**^ [0.00] | -1.69^**^ [0.00] | -0.42 [0.40] | -0.65 [0.22] | -1.59^**^ [0.00] |
| Vignetteset 7 (ref. 1) | -0.49 [0.27] | -0.69 [0.25] | -0.34 [0.57] | -0.13 [0.81] | -0.87 [0.18] |
| Vignetteset 8 (ref. 1) | -0.61 [0.16] | -1.47^*^ [0.03] | 0.14 [0.78] | -0.25 [0.65] | -0.97 [0.15] |
| Intercept | 4.44^***^ [0.00] | 5.14^***^ [0.00] | 3.15^***^ [0.00] | 3.95^***^ [0.00] | 4.52^***^ [0.00] |
| Respondent Level Variance | 2.23^***^ [0.00] | 2.21^***^ [0.00] | 2.03^***^ [0.00] | 2.13^***^ [0.00] | 2.20^***^ [0.00] |
| Vignette Level Variance | 2.93^***^ [0.00] | 2.53^***^ [0.00] | 3.27^***^ [0.00] | 2.60^***^ [0.00] | 3.21^***^ [0.00] |
| N (Vignettes) | 1544 | 792 | 752 | 792 | 752 |
| N (Respondents) | 193 | 99 | 94 | 99 | 94 |

*Note*: Stated Likeliness of Hiring the Candidate (0-10). Regression Models with main effects and controls.

Standard Errors are shown in Brackets.

* 𝑝 < 0.05, ** 𝑝 < 0.01, *** 𝑝 < 0.001

### **Table A.4**: Regression Models with Main Effects, England

|  | M1b: Combined | M2b: HRM | M3b: Software | 4b: Early Career | M5b: Late Career |
| --- | --- | --- | --- | --- | --- |
| Cultural Capital (ref. lower) | -0.04 [0.48] | -0.06 [0.45] | -0.02 [0.84] | 0.05 [0.55] | -0.13 [0.09] |
| Education Level (ref. Vocational) | 0.61^***^ [0.00] | 0.59^***^[0.00] | 0.63^***^ [0.00] | 0.60^***^[0.00] | 0.61^***^[0.00] |
| Education Specificity (ref. General) | 0.68^***^ [0.00] | 0.81^***^ [0.00] | 0.51^***^ [0.00] | 0.57^***^ [0.00] | 0.79^***^ [0.00] |
| Training | 0.74^***^ [0.00] | 0.64^***^ [0.00] | 0.86^***^ [0.00] | 0.70^***^ [0.00] | 0.78^***^ [0.00] |
| Work Experience in Field | 2.43^***^ [0.00] | 2.58^***^ [0.00] | 2.23^***^ [0.00] | 2.58^***^ [0.00] | 2.27^***^ [0.00] |
| Gender (ref. Men) | 0.06 [0.37] | 0.00 [0.98] | 0.14 [0.17] | 0.07 [0.43] | 0.06 [0.61] |
| Early/Late Career (ref. Early) | -0.20 [0.39] | 0.00 [1.00] | -0.38 [0.26] |  |  |
| Software (ref. HRM) | 0.66^**^ [0.00] |  |  | 0.94^**^ [0.01] | 0.36 [0.25] |
| Vignetteset 2 (ref. 1) | 0.10 [0.79] | 0.32 [0.56] | -0.07 [0.89] | -0.08 [0.88] | 0.41 [0.44] |
| Vignetteset 3 (ref. 1) | -0.55 [0.16] | -0.50 [0.39] | -0.59 [0.27] | -0.53 [0.92] | -0.39 [0.41] |
| Vignetteset 4 (ref. 1) | 0.04 [0.92] | -0.02 [0.97] | 0.21 [0.73] | 0.07 [0.92] | 0.10 [0.84] |
| Vignetteset 5 (ref. 1) | 0.22 [0.56] | 0.48 [0.37] | -0.03 [0.73] | 0.09 [0.87] | 0.46 [0.40] |
| Vignetteset 6 (ref. 1) | 0.20 [0.62] | 0.43 [0.44] | -0.11 [0.84] | 0.72 [0.19] | -0.58 [0.23] |
| Vignetteset 7 (ref. 1) | -0.36 [0.42] | -0.48 [0.46] | -0.02 [0.97] | -0.07 [0.88] | -0.62 [0.45] |
| Vignetteset 8 (ref. 1) | -0.30 [0.46] | 0.41 [0.45] | -1.12^*^ [0.04] | 0.81 [0.18] | -0.94^*^ [0.05] |
| Intercept | 3.49^***^ [0.00] | 3.16^***^[0.00] | 4.45^***^ [0.00] | 3.13^***^ [0.00] | 3.55^***^ [0.00] |
| Respondent Level Variance | 1.91^***^ [0.00] | 2.09^***^[0.00] | 1.49^*^ [0.04] | 2.01^***^ [0.00] | 1.49^*^ [0.02] |
| Vignette Level Variance | 3.06^***^ [0.00] | 2.92^***^ [0.00] | 3.19^***^ [0.00] | 2.81^***^ [0.00] | 3.29^***^ [0.00] |
| N (Vignettes) | 1368 | 768 | 600 | 696 | 672 |
| N (Respondents) | 171 | 96 | 75 | 87 | 84 |

*Note*: Stated Likeliness of Hiring the Candidate (0-10). Regression Models with main effects and controls.

Standard Errors are shown in Brackets.

* 𝑝 < 0.05, ** 𝑝 < 0.01, *** 𝑝 < 0.001

### **Table A.5**: Regression Models with Interaction Effects, Germany

|  | M6a: combined | M7a: HRM | M8a: Software | M9a: Early Career | M10a: Late Career |
| --- | --- | --- | --- | --- | --- |
| Cultural Capital (ref. lower) | -0.31^*^ | -0.20 | -0.38^*^ | -0.02 | -0.61^**^ |
|  | [0.14] | [0.21] | [0.19] | [0.19] | [0.20] |
| Education Level (ref. Vocational) | 0.24^*^ | 0.19 | 0.31 | 0.34^**^ | 0.14 |
|  | [0.10] | [0.12] | [0.16] | [0.13] | [0.15] |
| CC * Level | 0.26^*^ | 0.29 | 0.21 | 0.13 | 0.40^*^ |
|  | [0.13] | [0.17] | [0.19] | [0.18] | [0.18] |
| Education Specificity (ref. General) | 0.98^***^ | 0.87^***^ | 1.11^***^ | 1.01^***^ | 0.96^***^ |
|  | [0.09] | [0.12] | [0.14] | [0.13] | [0.14] |
| CC * Specificity | 0.21 | 0.11 | 0.29 | 0.23 | 0.19 |
|  | [0.12] | [0.16] | [0.17] | [0.17] | [0.17] |
| Training | 0.44^***^ | 0.29^*^ | 0.62^***^ | 0.56^***^ | 0.33^**^ |
|  | [0.09] | [0.11] | [0.15] | [0.14] | [0.13] |
| CC * Training | 0.23 | 0.34^*^ | 0.08 | -0.06 | 0.53^**^ |
|  | [0.13] | [0.15] | [0.21] | [0.19] | [0.17] |
| Work Experience in Field | 2.41^***^ | 2.15^***^ | 2.68^***^ | 2.84^***^ | 1.95^***^ |
|  | [0.18] | [0.23] | [0.28] | [0.23] | [0.28] |
| CC * Experience | 0.22^*^ | 0.16 | 0.28 | 0.04 | 0.41^**^ |
|  | [0.10] | [0.13] | [0.16] | [0.14] | [0.15] |
| Gender (ref. Men) | 0.05 | 0.17^*^ | -0.07 | 0.08 | 0.02 |
|  | [0.06] | [0.08] | [0.08] | [0.09] | [0.07] |
| Late Career (ref. Early) | -0.49^*^ | -0.70^*^ | -0.30 |  |  |
|  | [0.23] | [0.32] | [0.33] |  |  |
| Software (ref. HRM) | -0.58^*^ |  |  | -0.79^*^ | -0.40 |
|  | [0.23] |  |  | [0.32] | [0.33] |
| Vignetteset 2 (ref. 1) | -0.47 | -0.43 | -0.40 | 0.04 | -1.02^*^ |
|  | [0.39] | [0.48] | [0.56] | [0.60] | [0.45] |
| Vignetteset 3 (ref. 1) | -0.87^*^ | -1.53^**^ | -0.26 | -0.44 | -1.36^**^ |
|  | [0.41] | [0.52] | [0.57] | [0.58] | [0.53] |
| Vignetteset 4 (ref. 1) | -0.78 | -1.08 | -0.51 | 0.09 | -1.69^*^ |
|  | [0.52] | [0.71] | [0.75] | [0.56] | [0.85] |
| Vignetteset 5 (ref. 1) | -0.93^*^ | -1.67^**^ | -0.13 | -0.63 | -1.30^*^ |
|  | [0.41] | [0.56] | [0.58] | [0.63] | [0.52] |
| Vignetteset 6 (ref. 1) | -1.10^**^ | -1.69^**^ | -0.42 | -0.65 | -1.59^**^ |
|  | [0.37] | [0.52] | [0.50] | [0.53] | [0.51] |
| Vignetteset 7 (ref. 1) | -0.49 | -0.69 | -0.34 | -0.13 | -0.87 |
|  | [0.44] | [0.61] | [0.61] | [0.57] | [0.65] |
| Vignetteset 8 (ref. 1) | -0.61 | -1.47^*^ | 0.14 | -0.25 | -0.97 |
|  | [0.43] | [0.70] | [0.51] | [0.55] | [0.68] |
| Intercept | 4.67^***^ | 5.36^***^ | 3.37^***^ | 4.04^***^ | 4.89^***^ |
|  | [0.34] | [0.44] | [0.43] | [0.43] | [0.45] |
| Respondent Level Variance | 2.23^***^ | 2.21^***^ | 2.03^***^ | 2.13^***^ | 2.21^***^ |
|  | [0.13] | [0.21] | [0.16] | [0.15] | [0.20] |
| Vignette Level Variance | 2.92^***^ | 2.51^***^ | 3.25^***^ | 2.59^***^ | 3.17^***^ |
|  | [0.10] | [0.13] | [0.14] | [0.12] | [0.15] |
| N Vignettes | 1544 | 792 | 752 | 792 | 752 |
| N Respondents | 193 | 99 | 94 | 99 | 94 |

*Note*: Stated Likeliness of Hiring the Candidate (0-10). Regression Models with Interactions and Controls.

Standard Errors are shown in Brackets.

* 𝑝 < 0.05, ** 𝑝 < 0.01, *** 𝑝 < 0.001

### **Table A.6:** Regression Models with Interaction Effects, Germany

|  | M6b: combined | M7b: HRM | M8b: Software | M9b: Early Career | M10b: Late Career |
| --- | --- | --- | --- | --- | --- |
| Cultural Capital (ref. lower) | -0.14 | -0.32 | 0.10 | -0.24 | -0.02 |
|  | [0.16] | [0.22] | [0.24] | [0.25] | [0.21] |
| Education Level (ref. Vocational) | 0.71^***^ | 0.72^***^ | 0.71^***^ | 0.73^***^ | 0.70^***^ |
|  | [0.12] | [0.18] | [0.16] | [0.16] | [0.18] |
| CC * Level | -0.21 | -0.28 | -0.16 | -0.24 | -0.20 |
|  | [0.16] | [0.22] | [0.24] | [0.23] | [0.23] |
| Education Specificity (ref. General) | 0.67^***^ | 0.75^***^ | 0.56^***^ | 0.43^**^ | 0.91^***^ |
|  | [0.11] | [0.16] | [0.16] | [0.15] | [0.16] |
| CC * Specificity | 0.01 | 0.11 | -0.11 | 0.27 | -0.26 |
|  | [0.14] | [0.20] | [0.22] | [0.18] | [0.22] |
| Training | 0.67^***^ | 0.50^**^ | 0.89^***^ | 0.61^***^ | 0.75^***^ |
|  | [0.12] | [0.18] | [0.16] | [0.17] | [0.17] |
| CC * Training | 0.15 | 0.29 | -0.05 | 0.19 | 0.09 |
|  | [0.15] | [0.21] | [0.20] | [0.21] | [0.22] |
| Work Experience in Field | 2.30^***^ | 2.39^***^ | 2.19^***^ | 2.40^***^ | 2.20^***^ |
|  | [0.18] | [0.22] | [0.30] | [0.24] | [0.27] |
| CC * Experience | 0.26^*^ | 0.39^*^ | 0.09 | 0.36^*^ | 0.15 |
|  | [0.13] | [0.19] | [0.15] | [0.18] | [0.18] |
| Gender (ref. Men) | 0.07 | 0.01 | 0.14 | 0.09 | 0.06 |
|  | [0.07] | [0.10] | [0.10] | [0.09] | [0.11] |
| Late Career (ref. early) | -0.20 | 0.00 | -0.38 |  |  |
|  | [0.24] | [0.32] | [0.33] |  |  |
| Software (ref. HRM) | 0.66^**^ |  |  | 0.94^**^ | 0.36 |
|  | [0.23] |  |  | [0.34] | [0.31] |
| Vignetteset 2 (ref. 1) | 0.10 | 0.32 | -0.07 | -0.08 | 0.41 |
|  | [0.39] | [0.56] | [0.53] | [0.56] | [0.53] |
| Vignetteset 3 (ref. 1) | -0.55 | -0.50 | -0.59 | -0.53 | -0.39 |
|  | [0.39] | [0.57] | [0.54] | [0.59] | [0.47] |
| Vignetteset 4 (ref. 1) | 0.04 | -0.02 | 0.21 | 0.07 | 0.10 |
|  | [0.43] | [0.58] | [0.61] | [0.72] | [0.49] |
| Vignetteset 5 (ref. 1) | 0.22 | 0.48 | -0.03 | 0.09 | 0.46 |
|  | [0.39] | [0.54] | [0.56] | [0.54] | [0.55] |
| Vignetteset 6 (ref. 1) | 0.20 | 0.43 | -0.11 | 0.72 | -0.58 |
|  | [0.40] | [0.55] | [0.54] | [0.55] | [0.48] |
| Vignetteset 7 (ref. 1) | -0.36 | -0.48 | -0.02 | -0.07 | -0.62 |
|  | [0.45] | [0.65] | [0.49] | [0.45] | [0.82] |
| Vignetteset 8 (ref. 1) | -0.30 | 0.41 | -1.12^*^ | 0.81 | -0.94^*^ |
|  | [0.41] | [0.54] | [0.56] | [0.61] | [0.48] |
| Intercept | 3.54^***^ | 3.29^***^ | 4.39^***^ | 3.26^***^ | 3.49^***^ |
|  | [0.34] | [0.45] | [0.40] | [0.47] | [0.37] |
| Respondent Level Variance | 1.92^***^ | 2.09^***^ | 1.49^*^ | 2.02^***^ | 1.50^*^ |
|  | [0.12] | [0.16] | [0.15] | [0.17] | [0.12] |
| Vignette Level Variance | 3.05^***^ | 2.90^***^ | 3.18^***^ | 2.79^***^ | 3.28^***^ |
|  | [0.11] | [0.12] | [0.19] | [0.13] | [0.17] |
| N Vignettes | 1368 | 768 | 600 | 696 | 672 |
| N Respondents | 171 | 96 | 75 | 87 | 84 |

*Note*: Stated Likeliness of Hiring the Candidate (0-10). Regression Models with Interactions and Controls.

Standard Errors are shown in Brackets.

* 𝑝 < 0.05, ** 𝑝 < 0.01, *** 𝑝 < 0.001

### **Table A.7:** T-Tests on Respondent-Level Indicators in HRM in England between Prolific- and other Respondents

|  | Prolific | Others | Difference | p-value (T-test) |
| --- | --- | --- | --- | --- |
| Typical Income for HR Manager Position | 44400 | 41049.18 | 3350.82 | 0.0839 |
| Respondent Education (University Educated) | 0.76 | 0.82 | 0.056 | 0.26 |
| Higher Education in Firm | 1.51 | 1.66 | 0.15 | 0.26 |
| Firm Size | 2780.74 | 3223.09 | -442.34 | 0.58 |
| Importance of Leadership | 4.31 | 4.2 | 0.11 | 0.77 |
| Importance of Social Skills | 4.23 | 4.17 | -0.06 | 0.67 |
